# Case of Pseudo-Membranous Laryngitis

**Published:** 1849-12

**Authors:** 


*Case of Pseudo-membranous Laryngitis.* By Dr. W. CHEW VAN BIBBER, of Baltimore,

Recoveries from pseudo-membranous laryngitis are so rare that each is worthy of being remembered.

The following case is interesting on account of the urgency of the symptoms, the unmistakeable character of the disease, and the promptitude of the relief.

Hugh Brady, aged 3 years and 2 months, was observed, on the 11th of January, to have catarrh, with cough and hoarseness.

On the 13th, Dr. J. L. Webster was called; found him with high fever, difficult respiration, ringing, croupy cough, voice a whisper, throat inflamed, tonsils enlarged; no membranous exudation on tonsils or fauces.

On 14th. Cauterized the throat with water ℥i, nit. argent. grs. xiv. Prescribed calomel and emetics, with a blister on the sternum. This treatment was continued until I saw him in consultation, on the 23d, the 12th day of the disease.

*Symptoms.*—Respiration 32, forced; alæ nasi expanded and mouth open; loud blowing and hissing sound in inspiration and expiration, but louder in inspiration; head thrown back, buried deep in the pillow; laborious heaving of chest; countenance of fearful anxiety; eyes open and glassy; pupils contracted; profuse perspiration over head, chest, and upper extremities; legs and feet dry and hot; pulse full and fast, 140.

Sibilant and mucous rales on both sides; if any other sounds existed, they were masked by the loud blowing sounds in the larynx and trachea. Tongue and mouth parched and dry. No false membrane could be discovered on the throat, by the red light of a candle.

Having acquired some dexterity with the probang, we attempted to pass Dr. Green's instrument into the larynx. Saw the epiglottis erect, but failed to get the sponge entirely through the cartilages. From the violent expulsive efforts produced, we concluded (which is no doubt true) a small portion of it dropped into the trachea. In a few moments there were expelled by these efforts, many fragments of false membrane, the longest of which was nearly two inches, ragged and detaching itself in water; was light colored and tough; we attempted to pass it again, and a third time with the same result. At the last attempt there was no more false membrane expelled.

In half an hour an emetic of sulph. cupri. Continue calomel with mercurial plaster to the throat.

24th.—No better. Pulse quicker and more contracted.

Used probang with the same results as before, three times in succession. The solution being, aq. ℥i; nit. arg. (crys.) ʒi. An emetic of alum ʒi, repeated. Suspend calomel.

25th.—Better.

26th.—Greatly relieved. Laryngeal stridulous sound almost gone, loud mucous and sonorous rale in both lungs, particularly the right. Profuse perspiration, and urine scanty. ℞. Mur. ammon. grs. iij; tinct. digitalis. ♏ss; ext. hyosciamus, gr. ¼. Every two hours in solution. Sponge the surface with a solution of salt and water, and frictions with warm flannel.

27th.—Better in every respect, and from this time he recovered.

It has been said (and with what justice you can determine) that 'physicians have been tinkering at the constitution for two thousand years, and but two specifics are recognized, viz., mercury and sulphur.' That the nitrate of silver may prove, when properly applied, a specific in pseudo-membranous laryngitis, I sincerely hope, and from the effects I have observed from it when applied to false membrane appearing on the tonsils and pharynx, I think it not improbable.—*Am. Journ. Med. Sciences.*

